# SLX1 Inhibition Enhances Olaparib Sensitivity by Impairing Homologous Recombination Repair in Breast Cancer

**DOI:** 10.3390/ijms262311621

**Published:** 2025-11-30

**Authors:** Jin-Young Kim, Jeeho Kim, In-Youb Chang, Sang-Gon Park, Ho Jin You, Young Jin Jeon, Jung-Hee Lee

**Affiliations:** 1 Laboratory of Genomic Instability and Cancer Therapeutics, Chosun University School of Medicine, 375 Seosuk-dong, Gwangju 61452, Republic of Korea; kkk423@chosun.ac.kr (J.-Y.K.); jeehokim@chosun.ac.kr (J.K.); hjyou@chosun.ac.kr (H.J.Y.); 2 Department of Cellular and Molecular Medicine, Chosun University School of Medicine, 375 Seosuk-dong, Gwangju 61452, Republic of Korea; 3 Department of Pharmacology, Chosun University School of Medicine, 375 Seosuk-dong, Gwangju 61452, Republic of Korea; 4 Department of Anatomy, Chosun University School of Medicine, 375 Seosuk-dong, Gwangju 61452, Republic of Korea; iyjang@chosun.ac.kr; 5 Department of Hemato-Oncology, Chosun University Hospital Internal Medicine, Gwangju 61453, Republic of Korea; sgpark@chosun.ac.kr

**Keywords:** SLX1, PARP inhibitors, homologous recombination (HR) repair, breast cancer

## Abstract

While PARP inhibitors like Olaparib are effective against BRCA1-deficient breast cancers, their efficacy in BRCA1-proficient tumors depends on the functional status of homologous recombination (HR) repair. Here, we identify the structure-specific endonuclease SLX1 as a key regulator of HR and a determinant of Olaparib sensitivity in BRCA1-intact breast cancer. SLX1 is frequently upregulated in breast cancer and associated with poor prognosis. Functional studies revealed that SLX1 promotes RAD51-mediated HR repair of DNA double-strand breaks. Consequently, SLX1 depletion reduces HR efficiency, increases chromosomal instability, and sensitizes breast-proficient breast cancer cells to DNA-damaging agents, including camptothecin, ionizing radiation, and Olaparib. In contrast, SLX1 overexpression enhances DNA repair capacity and promotes Olaparib resistance. In vivo, SLX1 knockdown synergizes with Olaparib to suppress tumor growth in xenograft models. These findings establish SLX1 as a critical regulator of HR function in BRCA1-proficient breast cancer and a promising target for restoring PARP inhibitor sensitivity through induced HR deficiency.

## 1. Introduction

Breast cancer is the most prevalent type of malignancy as well as the major cause of cancer deaths in women worldwide [1]. Triple-negative breast cancer (TNBC), defined by the absence of ER, PR, and HER2, accounts for 10–15% of all cases and frequently involves defects in the BRCA1/2 DNA repair pathway [2,3,4,5,6]. Loss of BRCA1/2 functions impairs homologous recombination (HR), leaving tumor cells reliant on an alternative repair mechanism that is vulnerable to PARP inhibitor, which induces the accumulation of unrepaired DNA damage and cell death [7,8,9,10,11,12,13].

Although PARP inhibitors are for BRCA-mutated cancers, their activity in BRCA-proficient tumors is dependent on HR repair function rather than BRCA genotypes per se [9,10]. Therefore, within these BRCA-wild cells, PARP-induced DNA damage will only be toxic if there is a pre-existing deficiency in HR repair. This is precisely why functional biomarkers such as RAD51 foci formation are better indicators of response outcomes rather than static genomic defects [14,15]. The TBCRC-048 trial confirmed that Olaparib worked effectively in BRCA-normal breast cancers with PALB2 mutations that impair HR function, but failed in those with ATM/CHK2 mutations that leave HR intact [16]. This finding demonstrates that HR function, not BRCA status alone, determines PARP inhibitor sensitivity. Consequently, drugs that block HR activation regulators could restore PARP inhibitor effectiveness in resistant BRCA-normal breast cancers by creating a functionally HR-deficient state [17,18,19,20].

SLX1 (structure-specific endonuclease subunit SLX1) is a conserved GIY-YIG endonuclease that functions as the catalytic subunit of the SLX1-SLX4 complex. SLX1 is activated upon binding to SLX4, enabling it to cleave branched DNA structures [21,22,23,24]. In human cells, the SLX1-SLX4 complex resolves recombination and replication intermediates, such as Holliday junctions and replication forks, to maintain genome stability under genotoxic stress [25,26,27,28,29]. However, while their general functions are known, the specific contribution of SLX1 to the RAD51-mediated HR repair pathway, and whether its expression levels could function as a modulator of PARP inhibitor sensitivity, have not been investigated.

Here, we show that SLX1 is significantly upregulated in breast cancer and that high expression of SLX1 is associated with poor prognosis. We demonstrate that SLX1 promotes RAD51-mediated HR repair in breast cancer. Furthermore, we find that silencing SLX1 sensitizes cells to the PARP inhibitor Olaparib, whereas its upregulation is associated with Olaparib resistance, thereby identifying SLX1 as a potential therapeutic target.

## 2. Results

### 2.1. SLX1 Is Overexpressed in Breast Cancer, Particularly in Those with Poor Prognosis

**Figure 1:**
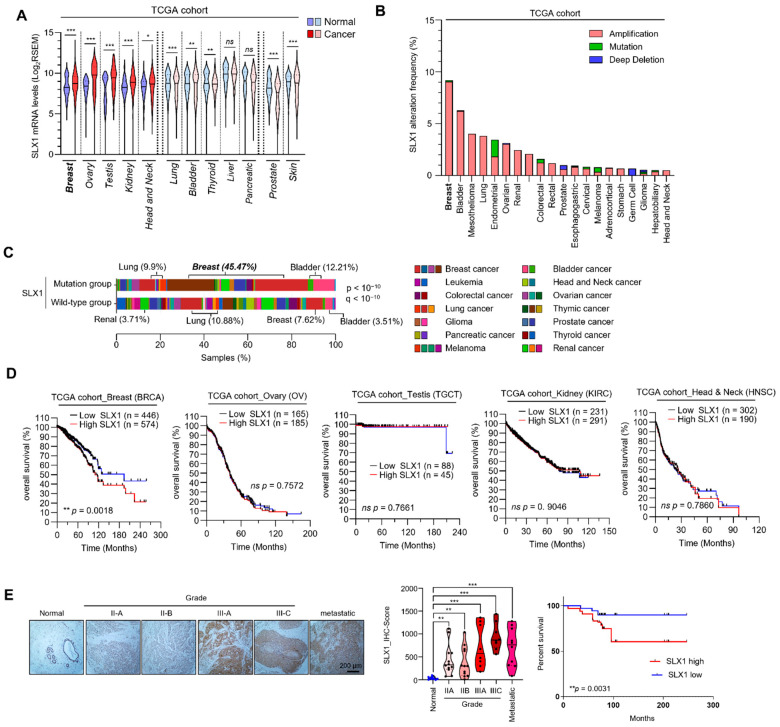
SLX1 is overexpressed in breast cancer cells. (**A**) SLX1 mRNA expression levels across 13 primary cancer types from the TCGA dataset are compared to normal tissue. Data presented as mean ± SEM. Statistical analysis is done using a two-tailed Student’s *t*-test (* *p* < 0.05, ** *p* < 0.01, *** *p* < 0.001, ns, not significant). (**B**) Pan-cancer analysis of SLX1 gene alterations and mutation frequencies in specific cancer types is conducted using TCGA data. (**C**) Distribution of SLX1 mutations in various cancers. Samples are divided into mutation and wild-type groups (*p* < 10^−10^, q < 10^−10^), with percentage of samples shown on x-axis. (**D**) Kaplan–Meier plot showing the association between SLX1mRNA expression levels and overall survival in breast (BRCA), ovary (OV), and testis (TGCT) cancer patients from the TCGA cohort. Log-rank test is used for statistical significance. (**E**) Immunohistochemistry (IHC) analysis shows SLX1 expression in normal breast tissue, tumors of varying grades (IIA, IIB, IIIA, IIIC), and metastatic breast cancer. Hematoxylin is a counterstain in IHC. Scale bar = 200 μm. Lower left panel is a quantification of SLX1 expression from a tissue microarray (TMA) containing 9 normal tissues, 12 grade IIA, 12 grade IIB, 8 grade IIIA, 8 grade IIIC, and 10 metastatic tumor samples. Analysis is done with a Mann–Whitney test (** *p* < 0.01, *** *p* < 0.001). Lower right panel is a Kaplan–Meier plot showing overall survival in patients with high vs. low SLX1 expression based on average IHC scores (log-rank test).

Through the analysis of the RNA sequencing (RNA-Seq) data obtained from The Cancer Genome Atlas (TCGA), we found that SLX1 is an upregulated gene in multiple cancers, including the highest expression observed in breast cancer (Figure 1A). SLX1 overexpression was observed across all major molecular subtypes of breast cancer. High SLX1 expression also correlated with a high frequency of genomic alterations, an occurrence most prominent in breast cancer, where gene amplification was the predominant alteration type (Figure 1B,C). In order to analyze the clinical value of SLX1 expression, we prepared a Kaplan–Meier survival analysis in the TCGA group. High SLX1 expression was significantly linked to poor overall survival in specifically breast cancer patients, while no such correlation was observed for other cancers such as ovary, testis, and kidney (Figure 1D and Figure S1). To confirm these results at the protein level, we performed tissue microarray (TMA) analysis, which showed significantly higher SLX1 protein expression in breast cancer tissues than in normal tissues. Moreover, higher SLX1 protein expression in cancer tissue increased progressively in higher grades of cancer, including metastatic lesions (Figure 1E, upper panel). Quantification of staining confirmed statistically significant differences across tumor grades (Figure 1E, lower left panel). Consistent with the TCGA analysis, as high SLX1 protein expression associated patients had a worse survival rate than those with low SLX1 protein expression (Figure 1E, lower right panel). Collectively, these findings indicate that SLX1 is substantially overexpressed in breast cancer and is associated with poor prognosis, suggesting that SLX1 is a promising therapeutic target.

### 2.2. SLX1 Promotes Homologous Recombination Repair of DNA Double-Strand Breaks

**Figure 2:**
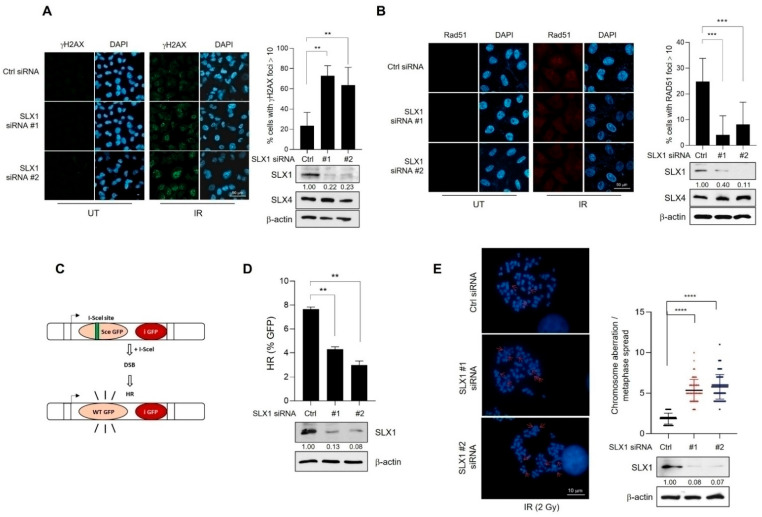
SLX1 is required for homologous recombination and genomic stability after DNA damage. (**A**,**B**) HeLa cells, either with or without an SLX1 knockdown, are irradiated with 10 Gy of IR and analyzed by indirect immunofluorescence with anti-γH2AX (**A**) or anti-RAD51 (**B**) antibodies at 20 and 6 h post-irradiation, respectively. DNA is counterstained with DAPI. Scale bar = 50 μm. The percentage of cells with more than ten RAD51 foci is indicated. SLX1 knockdown is confirmed by Western blot. SLX1 band intensities are quantified by densitometry using ImageJ v1.54p, and normalized to β-actin. Relative intensities are shown below SLX1 band. Mean ± SEM (n = 3). ** *p* < 0.01, *** *p* < 0.001, **** *p* < 0.0001. (**C**) A schematic representation of fluorescence-based assay used to evaluate HR-mediated DSB repair via II-SceI-mediated cleavage of the I-SceI site, which prerequisite for the production of functional green fluorescent protein (GFP). (**D**) HR-mediated DSB repair efficiency in DR-GFP-HeLa cells treated with either control or SLX1 siRNA as measured by FACS analysis. Data are shown as mean ± SEM (n = 3). ** *p* < 0.01. (**E**) Chromosomal aberrations induction in control and SLX1-depleted HeLa cells after exposure to a dose of 2 Gy of IR. Representative images of metaphase chromosome spreads and quantification are shown. Data are presented as mean ± SEM (n = 3). **** *p* < 0.0001, Mann–Whitney test. All experiments are independently repeated three times.

To address the involvement of SLX1 in DSB repair, we knocked down its expression in HeLa cells using two independent siRNAs. We first assessed DNA damage by monitoring γH2AX, a known marker for DSBs. Twenty hours after IR exposure, immunofluorescence revealed a significant accumulation of persistent γH2AX foci in SLX1-depleted cells compared to controls (Figure 2A). We then examined the recruitment of RAD51, a central HR component. IR-induced RAD51 foci formation was compromised in SLX1-silenced cells (Figure 2B). To confirm that these effects were specifically due to the loss of SLX1, we performed rescue experiments by reintroducing Flag-tagged SLX1 into SLX1-depleted cells. The re-expression of SLX1 effectively rescued both phenotypes, leading to a significant reduction in γH2AX foci and a restoration of RAD51 foci formation after IR (Figure S2A,B). Western blot analysis confirmed the knockdown was specific to SLX1, as levels of its binding partner, SLX4, were unaffected (Figure 2A,B). To directly measure the impact on specific repair pathways, we used established reporter assays. In a DR-GFP U2OS reporter system [30], SLX1 knockdown caused a pronounced reduction in HR efficiency (Figure 2C,D). In contrast, using an EJ5-GFP reporter [30], we found no significant difference in non-homologous end joining (NHEJ) activity between SLX1-knockdown and control cells (Figure S3A,B). These results indicate a specific requirement for SLX1 in the HR pathway. Given the importance of effective HR in maintaining chromosomal stability, we next investigated chromosomal aberrations following IR exposure. Microscopic analysis revealed a threefold increase in chromosomal aberrations per cell in the SLX1-knockdown cells as compared to the control group (Figure 2E). Collectively, these data demonstrate that SLX1 has a distinct role in HR-mediated DSB repair, where a lack of SLX1 compromises RAD51 recruitment, leading to reduced HR efficiency and chromosomal instability.

### 2.3. SLX1 Facilitates Effective DNA Double-Strand Break Repair in Breast Cancer Cells

**Figure 3:**
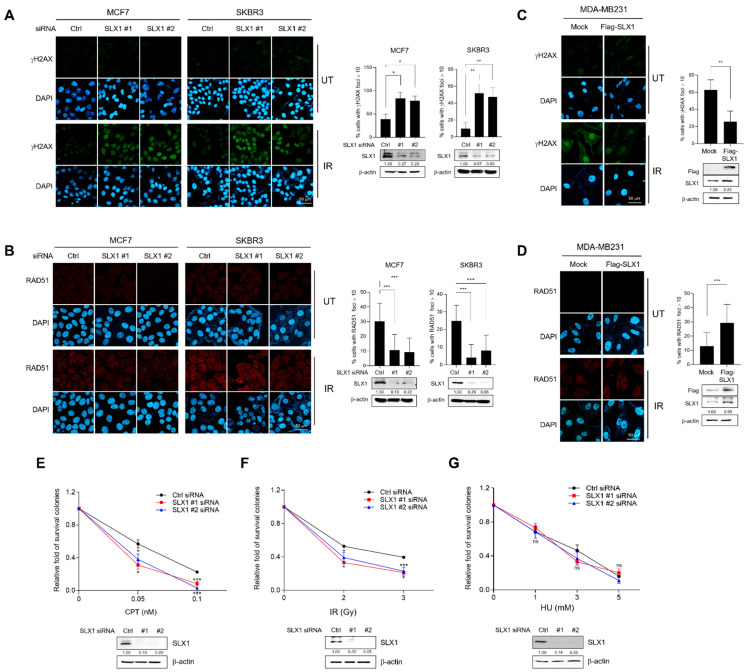
SLX1 contributes to double-strand break (DSB) repair in human breast cancer cells. (**A**,**B**) MCF7 and SK-BR3 cells with or without an SLX1 knockdown are irradiated with 10 Gy of ionizing radiation (IR) and fixed at various time points. Cells are immunostained with antibodies against γH2AX (**A**) or RAD51 (**B**), respectively, and DNA is counterstained with DAPI. Scale bar = 50 μm. Percentage of cells harboring more than ten γH2AX or RAD51 foci is shown. Data represent mean ± SEM. * *p* < 0.05, ** *p* < 0.01. (**C**,**D**) MDA-MB231 cells are transfected with either Flag-mock or Flag-SLX1. After irradiation of 10 Gy of IR, cells are fixed at the indicated time points. Immunofluorescence staining of cells is done with γH2AX (**C**) or RAD51 (**D**) antibodies, respectively. DNA is counterstained with DAPI. Histograms (right panels) represent the percentage of cells harboring more than ten γH2AX or RAD51 foci. Scale bars = 50 μm. Data are presented as mean ± SEM. ** *p* < 0.01, two-tailed Student’s *t*-test. (**E**–**G**) MCF7 cells are transfected with a control siRNA or two SLX1-targeting siRNAs. After 24 h, cells are replated and exposed for 14 days with camptothecin (CPT) (**E**), ionizing radiation (**F**), or hydroxyurea (HU) (**G**) at the indicated concentrations. Cell survival data are presented as mean ± SEM (n = 3). * *p* < 0.05, *** *p* < 0.001, ns, not significant. SLX1 knockdown in all data is confirmed by Western blot. All experiments is independently repeated three times.

We determined whether SLX1 is involved in regulating HR in breast cancer by examining the protein expression of SLX1 in various cancer and normal cancer cell lines. We found that SLX1 expression was barely detectable in the MCF-10A normal breast epithelial cell line but was high in the MCF-7 and SK-BR3 cell lines (Figure S4A,B). Based on these findings, we established SLX1-knockdown models in high-expressing MCF-7 and SK-BR3 cells. We also generated an SLX1-overexpression model in the MDA-MB-231 cell line, which expressed SLX1 at low levels. To evaluate the involvement of SLX1 in DNA damage repair via HR, we treated these cells with IR and monitored DSB repair. SLX1 knockdown in MCF7 and SKBR3 cells led to a significant increase in persistent γH2AX foci 20 h after IR, alongside a marked reduction in RAD51 foci formation (Figure 3A,B). Conversely, gain-of-function experiments confirmed this finding; overexpression of Flag-SLX1 in MDA-MB231 cells produced the opposite phenotype, with fewer γH2AX foci and increased RAD51 foci following IR (Figure 3C,D). Furthermore, neither the knockdown nor the overexpression of SLX1 significantly altered cell cycle progression (Figure S5A,B). These data demonstrate that SLX1 is involved in the efficient repair of DSBs through the recruitment of RAD51, a function independent of cell cycle changes. To determine how SLX1-mediated repair affects cell viability, we performed clonogenic survival assays in SLX1-knockdown cells treated with different DNA-damaging agents. SLX1 knockdown led to substantially lower cell survival after treatment with Camptothecin (CPT) or IR, both of which cause DSBs (Figure 3E,F and Figure S6A,B). In contrast, treatment with hydroxyurea (HU), which stalls replication forks, had only a minor effect on viability (Figure 3G and Figure S6C). Collectively, these findings indicate SLX1’s primary importance in the survival of breast cancer cells involving the HR-mediated repair of DSBs repair, rather than the resolution of stalled replication forks.

### 2.4. SLX1 Modulates Sensitivity to Olaparib in Breast Cancer Cells

**Figure 4:**
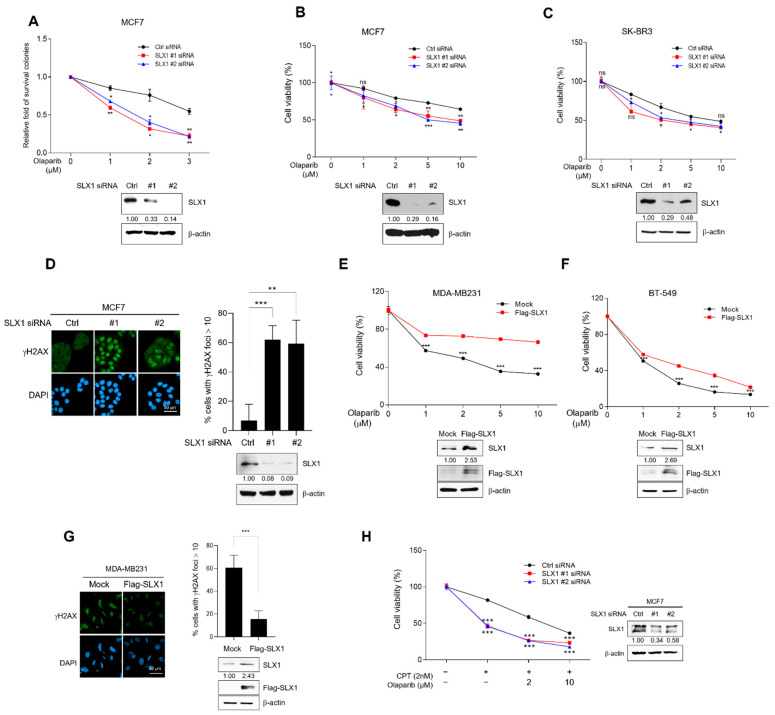
SLX1 modulates Olaparib sensitivity in breast cancer cells. (**A**) MCF7 cells with either a control or SLX1 knockdown are treated with a range of Olaparib concentrations for 48 h. After 14 days, a clonal survival analysis is done. Data are shown as mean ± SEM (n = 3). * *p* < 0.05, ** *p* < 0.01. (**B**,**C**) MCF7 (**B**) and SK-BR3 (**C**) cells are transfected with control siRNA or SLX1-specific siRNAs. After 24 h, Olaparib treatment is done for 48 h, and cell viability is assessed by MTT assay. Results are shown as mean ± SEM from three independent experiments. ns, not significant, * *p* < 0.05, ** *p* < 0.01, *** *p* < 0.001, ns, not significant. (**D**) Control and SLX1-knockdown MCF7 cells are treated with 10 μM Olaparib for 24 h and immunostained with anti-γH2AX antibody. DNA is counterstained with DAPI. Scale bar = 50 μm. Quantification shows the percentage of cells with >10 γH2AX foci. Data represent mean ± SEM. ** *p* < 0.01, *** *p* < 0.001. (**E**,**F**) MDA-MB231 (**E**) and BT-549 (**F**) cells are transfected with either control vector (Flag-mock) or Flag-tagged SLX1 (Flag-SLX1) for 24 h, then treated with Olaparib for 48 h. Cell viability is assessed by MTT assay. Data represent mean ± SEM from three independent experiments, *** *p* < 0.001. (**G**) MDA-MB231 cells transfected with Flag-mock or Flag-SLX1 are treated with 10 μM Olaparib for 24 h and immunostained with anti-γH2AX antibody. DNA is counterstained with DAPI. Scale bar = 50 μm. Quantification shows the percentage of cells with >10 γH2AX foci. Data represent mean ± SEM (n = 3). *** *p* < 0.001. (**H**) MCF7 cells are transfected with either control or SLX1-specific siRNAs for 24 h, then treat with the indicated concentrations of Olaparib in combination with 2 nM camptothecin (CPT) for an additional 48 h. Cell viability is assessed by MTT assay. Data present mean ± SEM (n = 3). *** *p* < 0.001. SLX1 knockdown in all data was confirmed by Western blot. All experiments are independently repeated three times.

Given SLX1’s role in HR-mediated DSBs repair, we hypothesized that SLX1 deficiency would increase sensitivity to the PARP inhibitor, Olaparib. To address this, we first performed clonogenic survival assays, which demonstrated a significantly lower survival fraction for SLX1-depleted MCF-7 cells compared to controls following Olaparib treatment. (Figure 4A). We confirmed this effect in additional cell lines using MTT viability assays, where both SLX1-knockdown MCF7 and SK-BR3 cells exhibited strongly reduced viability after Olaparib treatment (Figure 4B,C). To understand the molecular basis of this enhanced sensitivity, we assessed DNA damage levels. Immunofluorescence analysis revealed a marked increase in persistent γH2AX foci in SLX1-knockdown MCF7 cells 20 h post-Olaparib treatment (Figure 4D), suggesting that the loss of SLX1 reduces DNA damage repair activity in response to Olaparib treatment. Conversely, gain-of-function experiments supported this conclusion. MDA-MB-231 and BT-549 cells overexpressing Flag-tagged SLX1 exhibited markedly higher viability and substantially fewer γH2AX foci after Olaparib treatment (Figure 4E–G). We next investigated whether SLX1 modulates the efficacy of Olaparib in combination with other DNA-damaging drugs. The combination of Olaparib and CPT in SLX1-knockdown cells resulted in significantly increased cytotoxicity compared to monotherapy with either agent (Figure 4H and Figure S7A,B). Collectively, these data suggest that SLX1 expression serves as a predictive biomarker of PARP inhibitor responsiveness and that targeting SLX1 represents an effective strategy to enhance the efficacy of PARP inhibitors in breast cancer.

### 2.5. SLX1 Knockdown Increases Olaparib-Mediated Apoptosis in Breast Cancer Cells

**Figure 5:**
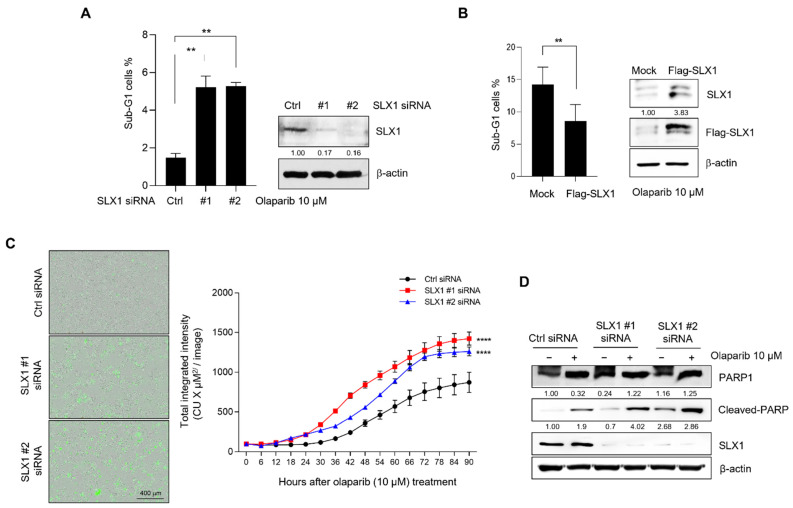
SLX1 is involved in Olaparib-induced apoptosis in breast cancer cells. (**A**,**B**) Control and SLX1-knockdown MCF7 cells (**A**), as well as MDA-MB231 cells that are transfected with either a control vector (Flag-mock) or SLX1-overexpressing vector (Flag-SLX1) (**B**), are maintained at 37 °C, 5% CO_2_ for 24 h. After that, the cells are then treated with Olaparib (10 μM) for 48 h before analysis of sub-G1 cells by flow cytometry. Data present mean ± SEM (n = 3). ** *p* < 0.01. SLX1 knockdown is confirmed by Western blot. (**C**) Apoptosis in control and SLX1-knockdown MCF7 cells is assessed using live-cell imaging after Annexin V staining. Data represent mean total integrated intensity ± SEM from three independent experiments. **** *p* < 0.0001. (**D**,**E**) MCF7 cells (**D**) with or without an SLX1 knockdown and MDA-MB231 cells. All experiments are independently repeated three times.

To assess if the increased Olaparib sensitivity in SLX1-depleted cells results from increased apoptosis, we first quantified the apoptotic sub-G1 population using flow cytometry. Following Olaparib exposure, we observed a significantly greater percentage of sub-G1 cells in SLX1-depleted populations compared to controls (Figure 5A). The sub-G1 fraction increased nearly threefold, indicating that SLX1 ablation enhances Olaparib-induced apoptosis. As expected, SLX1 overexpression in MCF7 cells produced the opposite effect, leading to an approximately 40% reduction in the sub-G1 populations after Olaparib treatment (Figure 5B).To further characterize the apoptosis dynamics, we performed live-cell imaging with Annexin V staining to detect the externalization of phosphatidylserine, an early apoptotic marker. The Annexin V signal appeared more rapidly and reached a greater intensity in SLX1-knockdown cells compared to controls following Olaparib exposure (Figure 5C). We confirmed these findings at the molecular level by assessing the cleavage of PARP1, a hallmark of apoptosis. While cleaved PARP1 was detected in both control and SLX1-depleted cells at 48 h post-treatment, its levels were notably higher in the SLX1-depleted cells (Figure 5D). Taken together, these findings suggest that SLX1 depletion enhances the therapeutic efficacy of Olaparib in breast cancer by promoting apoptosis.

### 2.6. SLX1 Knockdown Enhances Olaparib’s Antitumor Effects in a Breast Cancer Xenograft Model

**Figure 6:**
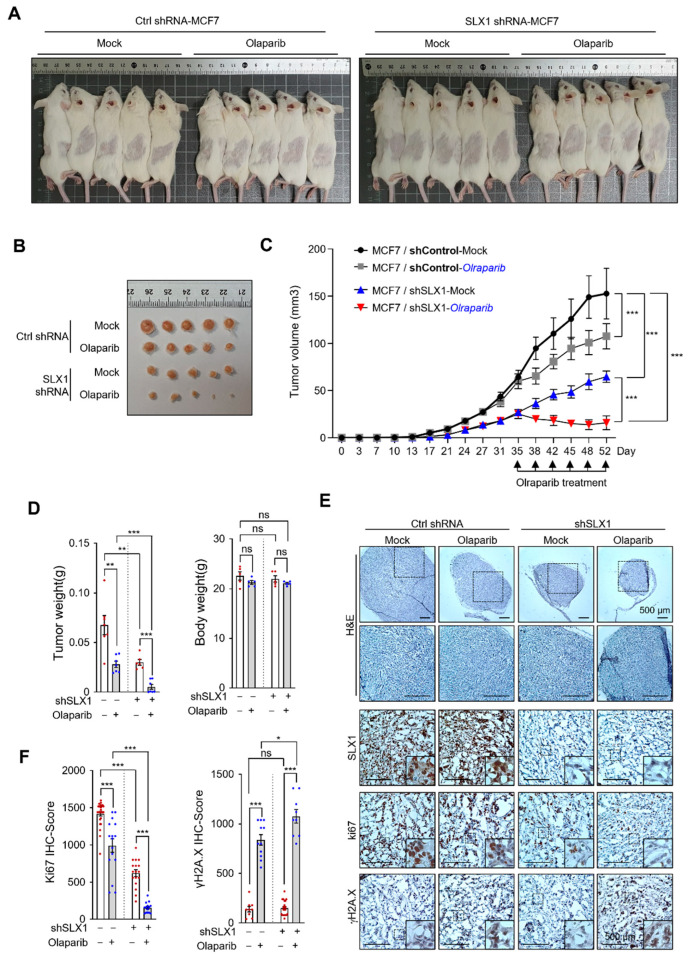
SLX1 depletion enhances the antitumor efficacy of Olaparib in a breast cancer xenograft model. (**A**,**B**) SCID mice (CB17/lcr-*Prkdc*^scid/scid^/Rj) were subcutaneously injected with control or SLX1-depleted MCF7 cells (n = 5 per group). Olaparib (50 mg/kg) or vehicle is administered intraperitoneally every 3 to 4 days starting 17 days post-transplantation. (**C**) Tumor volumes are recorded at the indicated time points. Data are shown as mean ± SEM. Statistical significance is assessed by two-way ANOVA (*** *p* < 0.001). (**D**) Tumor weights and body weights measured at the experimental endpoint. Data are presented as mean ± SEM. ** *p* < 0.01, *** *p* < 0.001, ns, not significant, two-tailed Student’s *t*-test. (**E**) Representative images of hematoxylin and eosin (H&E) staining and immunohistochemical staining for SLX1, Ki67, and γH2AX in MCF7 xenograft tumors. Scale bar = 500 μm. (**F**) Quantification of Ki67-positive and γH2AX-positive cells in tumor sections from treatment group. Data represent mean ± SEM. * *p* < 0.05, *** *p* < 0.001, ns, not significant, two-tailed Student’s *t*-test.

To validate our in vitro findings, we investigated whether SLX1 depletion enhances Olaparib efficacy in vivo. We established xenograft tumors in SCID mice using either control or SLX1-knockdown MCF-7 cells. Once the tumors reached a measurable size, mice were randomly assigned to four groups: control + vehicle, control + Olaparib, SLX1-knockdown + vehicle, and SLX-knockdown + Olaparib. Treatments were administered every 3–4 days for 52 days. This experimental design enabled the evaluation of SLX1 depletion and Olaparib treatment, both individually and in combination, on tumor growth (Figure 6A–C). Tumors in the control + vehicle group grew unabated throughout the study. Olaparib monotherapy had only modest tumor-suppressive effects, while SLX1-depletion alone also resulted in diminished growth compared to control tumors. Most strikingly, the combination of SLX1 depletion and Olaparib therapy profoundly suppressed tumor development, resulting in significantly smaller tumors than in all other groups. Quantitative analysis of tumor weights at the study’s endpoint confirmed these observations (Figure 6D). To understand the underlying mechanisms, we performed immunohistochemical analysis on the tumor tissues. Staining for Ki67, a proliferation marker, revealed that the combination therapy significantly reduced Ki67 levels compared to all other groups (Figure 6E,F). Concurrently, tumors from the combination therapy group showed significantly higher levels of γH2AX staining (Figure 6E,F), indicating persistent DNA damage. These in vivo findings corroborate our in vitro findings and demonstrate that SLX1 depletion enhances the antitumor efficacy of Olaparib in breast cancer.

## 3. Discussion

In this study, we not only identified SLX1 as a new factor in HR repair, but we also found a new target strategy that could improve the efficacy of PARP inhibitors in treating breast cancer. We found that SLX1 is significantly overexpressed in breast cancer tissues, where its expression correlates with higher tumor grade, metastasis, larger tumor size, and poor patient prognosis. Mechanistically, we demonstrate that SLX1 is crucial for HR-dependent DSB repair by facilitating RAD51 recruitment. Moreover, we established that SLX1 deficiency sensitizes breast cancer cells to the PARP inhibitor Olaparib treatment, while its overexpression confers resistance, highlighting a potent synthetic lethal interaction.

While PARP inhibitors show dramatic effects in BRCA1/2mutant tumors, their broader clinical application is limited by both primary and acquired resistance [9,31]. A key resistance mechanism involves restoration of HR repair, frequently through BRCA1/2 reversion mutations or altered expression of other HR factors [32,33,34]. For instance, suppressing RAD51 or inhibiting its activators, such as LRRK2, enhances PARP inhibitor sensitivity and reduces the emergence of resistance [35,36,37]. Similarly, ATR inhibition potentiates the cytotoxicity of PARP inhibitors in certain BRCA2-mutated tumor cells [17]. Our data align with this paradigm, uncovering SLX1 as a critical regulator of HR-dependent DSB repair. SLX1 depletion results in reduced RAD51 foci formation, decreased HR efficiency, and increased sensitivity to CPT and IR. Mechanistically, PARP inhibition prevents SSB repair, leading to accumulation of replication-associated DSBs. While HR-proficient cells resolve these lesions, SLX1-deficient cells fail to do so, leading to an accumulation of unrepaired DNA damage. This is demonstrated by the increased γH2AX foci and enhanced apoptotic response in these cells following Olaparib treatment. These findings suggest that targeting SLX1 to impair HR represents a promising strategy to enhance PARP inhibitor efficacy in breast cancer and potentially delay resistance development. A key strength of this study is its broad scope, integrating clinical data, comprehensive mechanistic studies, and in vivo validation. The correlation of SLX1 expression with clinical outcomes, established through TCGA and MTA dataset analyses, underscores the clinical relevance of our findings. Notably, our xenograft model provides robust preclinical evidence that combined SLX1 depletion and PARP inhibition produce synergistic tumor suppression, supporting the translational potential of this strategy. From a clinical perspective, these findings have two significant implications. First, SLX1 expression may serve as a predictive biomarker for PARP inhibitor response. Second, the synthetic lethal interaction between SLX1 deficiency and PARP inhibition suggests a promising therapeutic strategy: developing selective SLX1 inhibitors could overcome resistance and broaden the benefits of PARP inhibitors to a wider patient population.

In conclusion, this study establishes SLX1 as a crucial factor in HR-mediated DNA repair and confirms that its inhibition enhances Olaparib sensitivity. These findings provide a strong rationale for developing selective SLX1 inhibitors as a new treatment strategy to overcome PARP inhibitor resistance and improve outcomes for patients with breast cancer.

## 4. Materials and Methods

### 4.1. Cell Culture and Drug Treatment

Human cervix adenocarcinoma HeLa cells were cultured in Dulbecco’s Modified Eagle’s Medium (DMEM, Invitrogen, Waltham, MA, USA). Human breast cancer MCF7, SK-BR3, BT549, MDA-MB-231, MDA-MB-436, and MCF10A cells were grown in RPMI-1640 medium supplemented with 10% heat-inactivated fetal bovine serum (FBS), 100 units/mL penicillin, and 100 mg/mL streptomycin sulfate (Invitrogen). Cells were maintained in a humidified incubator containing 5% CO_2_ at 37 °C. All cells were obtained from the American Type Culture Collection (ATCC, Rockville, MD, USA). To induce DNA damage, growing cells were treated with the indicated dose of Camptothecin (CPT), Hydroxyurea (HU), and Olaparib for 48 h, or irradiated at the indicated Gy from a 137Cs source (Gamma cell 3000 Elan irradiator, Best Theratronics, Kanata, ON, Canada).

### 4.2. Antibodies and Reagents

SLX1 (TA331569, OriGene, Rockville, MD, USA), SLX4 (R3260-2, Abiocode, Agoura Hills, CA, USA), mouse anti-β-actin (sc-47778, Santa Cruz Biotechnology, Dallas, TX, USA), mouse anti-λH2AX (05-636-1, Millipore, Burlington, MA, USA), Rabbit anti-Rad51 (sc-8349, Santa Cruz Biotechnology), mouse anti-Flag (M2, Sigma, St Louis, MO, USA), PARP1 (ab191217, abcam, Cambridge, UK), Cleaved PARP #5625, cell signaling). Olaparib was purchased from Selleckchem (Houston, TX, USA). The Camptothecin, Hydroxyurea, and Oraparib were purchased from Sigma-Aldrich (Merck KGaA, Darmstadt, Germany).

### 4.3. Plasmid and RNA Interference and Transfection

The human pCMV-SLX1-Flag plasmid was purchased from Sino Biological (Sino Biological, Beijing, China). The two siRNA oligonucleotides were used for SLX1 knockdown: SLX1 #1 siRNA: 5′-GGCACACUGGACAGACCUGdTdT-3′ and SLX1 #2 siRNA: 5′ GGACAGACCUGCUGGAGAC -dTdT-3′. A scrambled siRNA was used as a negative control (Bioneer, Daejeon, Korea). Plasmids were introduced into cells via transfection using TurboFect (Thermo Fisher Scientific Laboratories, Middlesex, MA, USA) according to the manufacturer’s instructions. siRNAs were transfected at a final concentration of 50 nM using Lipofectamine RNAiMax (Invitrogen).

### 4.4. Generation of Stable SLX1 Knockdown Cells

For the generation of stable SLX1-depleted cell lines, oligonucleotides encoding the target sequence for SLX1-forward, 5′-GATCCGCACACTGGACAGACCTGCTGGAGATTCAAGAGATCTCCAGCAGGTCTGTCCAGTGTGCTTTTTTGGAAA-3′, and reverse, 5′-AGCTTTTCCAAAAAAGCACACTGGACAGACCTGCTGGAGATCTCTTGAATCTCCAGCAGGTCTGTCCAGTGTGCG-3′, were annealed and inserted into pSilencer2.1-U6-hygro vector (Ambion, Austin, TX, USA). MCF7 cells were transfected with control shRNA or SLX1 shRNA (pSilencer2.1-U6-hygro) using Turbofect and cultured in a selection medium containing 500 μg /mL G418 for 4–5 weeks. After selection, stable SLX1 knockdown clones were confirmed by Western blot analysis.

### 4.5. Quantitative Reverse Transcription Polymerase Chain Reaction (RT-qPCR) Analysis

Total RNA was isolated from cells using the Trizol Reagent (Invitrogen) according to the manufacturer’s instructions. RNA samples were subsequently treated with DNase I (Thermo Scientific) according to the manufacturer’s instructions. 1 µg of total or polyA+ RNA was reverse transcribed using Superscript III reverse transcriptase (Invitrogen) and either random hexamers (Macrogen, Seoul, Republic of Korea) or oligo-dT primers (Macrogen). Real-time quantitative PCR was performed with specific primers and SYBR Premix Ex Taq (Clontech, Mountain View, CA, USA) on a CFX96 (Bio-Rad, Hercules, CA, USA). The primer sequences include the SLX1 forward primer sequence, 5′-CTGTGCCAGATGGACACTGAGA-3′; the SLX1 reverse primer sequence, 5′-GTGGCACAGAAAAGAGGTAGGAG-3′; the GAPDH forward primer sequence, 5′-TGCACCACCAACTGCTTAGC-3′; and the GAPDH reverse primer sequence, 5′-GGCATGGACTGTGGTCATGAG-3′. The relative SLX1 mRNA expression levels were calculated using the 2−ΔΔCt method with GAPDH as the endogenous reference gene normalization.

### 4.6. Western Blot Analysis

Cells were lysed in RIPA buffer (50 mM Tris-HCl, pH 7.4, 150 mM NaCl, 1% Nonidet P-40, 0.5% sodium deoxycholate, 0.1% sodium dodecyl sulfate, 1 mM dithiothreitol, 1 mM phenylmethanesulfonylfluoride, 10 μg/mL leupeptin, and 10 μg/mL aprotinin) to extract proteins. Protein concentrations were measured using the Bio-Rad Protein Assay Kit. Equal amounts of proteins were separated on 10-12% SDS-PAGE gels and transferred onto a polyvinylidene difluoride (PVDF) membrane (PALL Life Sciences, Port Washington, NY, USA). Membranes were blocked with 5% nonfat milk in TBS-T (10 mM Tris-HCl (pH 7.4), 150 mM NaCl, and 0.1% Tween-20) for 1 h at room temperature, then incubated with appropriate primary antibodies overnight at 4 °C. After washing with TBS-T, membranes were incubated with peroxidase-conjugated secondary antibodies (1:5000, Jackson ImmunoResearch Inc., West Grove, PA, USA). After thorough washing with TBS-T and protein bands were visualized using an Enhanced Chemiluminescence (ECL) detection system (Intron Biotechnology, Seongnam-si, Republic of Korea). Uncropped blots are shown in a separate file of the uncropped original Western blot.

### 4.7. Immunofluorescence Analysis

Cells were plated onto glass coverslips and exposed to 10 Gy of ionizing radiation, then incubated at 37 °C for the indicated time points. After treatment, the cells were sequentially fixed with 4% paraformaldehyde for 10 min and ice-cold 98% methanol for 5 min, then permeabilized with 0.3% Triton X-100 for 15 min at room temperature. The coverslips were washed three times with PBS and blocked with freshly prepared blocking solution (5% bovine serum albumin in PBS) for 1 h at room temperature. Primary antibodies against RAD51 and γH2AX were applied, followed by incubation with Alexa Fluor 488 or Alexa Fluor 594-conjugated secondary antibodies (Molecular Probes, Waltham, MA, USA). Nuclei were counterstained with DAPI using Vectashield mounting medium. Fluorescence images were acquired using a confocal microscope (Zeiss LSM 510 Meta; Carl Zeiss, Oberkochen, Germany) and analyzed with Zeiss ZEN Image software (version 3.10) (Carl Zeiss). For foci quantification, cells with more than 10 foci were considered positive. The percentage of positive cells was determined from at least 100 cells per condition. Data are presented as the mean ± standard deviation from three independent experiments.

### 4.8. Immunohistochemistry

Immunohistochemistry (IHC) was conducted on breast cancer tissue microarrays (TMAs) obtained from Super Bio Chips (CBA6, Seoul, South Korea), which included tumor samples of varying grades along with adjacent normal tissues. For immunohistochemistry, antigen retrieval was performed by heating the slides in 1× antigen retrieval buffer (pH 9.0) (Abcam) at 95 °C for 15 min. Endogenous peroxidase activity was quenched with 3% H_2_O_2_ solution and was blocked prior to incubation with primary antibodies: anti-SLX1 (HPA047038 (1:200), Atlas antibodies, Stockholm, Sweden), anti-Ki67 (#9449 (1:100), Cell signaling technology, Danvers, MA, USA), and anti-γH2AX (#05-636 (1:400), Millipore, Darmstadt, Germany) overnight at 4 °C. This was followed by incubation with an HRP-conjugated secondary antibody for 1 h at room temperature, and subsequent incubation with DAB (3,3′-Diaminobenzidine) for 2 min. Subsequently, the slides were counterstained using Harris’s hematoxylin. Staining intensity was scored from 0 to 4, and the extent of staining was scored from 0% to 100%. The final H-score for each sample was calculated by multiplying the intensity score by the percentage of positive cells. All slides were evaluated independently by two pathologists who were blinded to clinical information.

### 4.9. MTT Assay

Cell viability was evaluated using the MTT assay. Briefly, cells (1 × 10^4^ per well) were seeded into 96-well plates in 100 μL of culture medium and incubated overnight at 37 °C in a humidified air containing 5% CO_2_. The following day, cells were treated with various concentrations of Olaparib and/or CPT for 48 h. MTT reagent was then added, and plates were incubated for 4 h at 37 °C. The resulting purple formazan crystals were solubilized in 100 μL of 0.04 N HCl in isopropanol per well. Absorbance was measured at 570 nm using a microplate spectrophotometer. Cell viability was expressed as the percentage relative to untreated control cells.

### 4.10. Clonal Survival Assay

Following treatment with IR, CPT, HU, or Olaparib, 5 × 10^2^ cells were plated in duplicate onto 60 mm dishes and incubated for 2–3 weeks at 37 °C in a humidified atmosphere with 5% CO_2_ to allow colony formation. Colonies were visualized by staining with 2% methylene blue in 50% ethanol and counted. The survival fraction was determined by comparing the plating efficiency of treated cells to that of untreated controls. Results are presented as the mean ± standard deviation of three independent experiments.

### 4.11. Annexin V Assay for Apoptosis

Cells were seeded at 4000–5000 cells per well in 96-well plates containing culture medium supplemented with 1 mM CaCl_2_. After 24 h, cells were treated with the indicated compounds, and Incucyte^®^ Annexin V Green Reagent (4642, Sartorius, Göttingen, Germany) was added according to the manufacturer’s instructions. Plates were placed in the Incucyte^®^ SX5 Live-Cell Analysis System (Sartorius), and phase-contrast and fluorescence images were automatically acquired every 6 h for up to 72 h. Green fluorescent object area (Annexin V-positive cells) was quantified and normalized to cell confluence using Incucyte Analysis Software (Version 2022B, Satorius). Data are presented as the mean integrated intensity ± SEM from three independent experiments.

### 4.12. Cell Cycle Assay by Flow Cytometry

The cell cycle was analyzed using flow cytometry. Briefly, cells were harvested by trypsinization, washed with PBS, and pelleted by centrifugation at 2000 rpm for 5 min. The cell pellet was resuspended in 1 mL of ice-cold 70% ethanol and fixed overnight. Fixed cells were washed twice with PBS, treated with 10 mg/mL RNase A (Thermo Fisher Scientific Inc., Waltham, MA, USA) for 30 min at 37 °C, and stained with 50 mg/mL Propidium Iodide (PI) for 15 min at room temperature in the dark. DNA content was analyzed using an Attune NxT flow cytometry (Invitrogen), and cell cycle distribution was determined using CellQuest Pro software (version 5.0.1) (BD Biosciences).

### 4.13. Chromosomal Aberration Analysis

HeLa cells transfected with control or SLX1 siRNA were treated with or without 2 Gy IR pf IR. At 24 h post-irradiation, cells were arrested in metaphase by the addition of 100 ng/mL colcemid (Sigma-Aldrich) for an additional 24 h. Cells were then harvested, pelleted by centrifugation, and gently resuspended in a hypotonic solution (40% of culture medium in distilled water) for 10 min at 37 °C. Following hypotonic treatment, cells were fixed in ice-cold methanol/acetic acid (3:1, *v*/*v*) for 30 min. After centrifugation and removal of the supernatant, cell pellets were resuspended in fresh fixative solution, dropped onto pre-cleaned glass slides, and air-dried overnight at room temperature. Slides were mounted with Vectashield mounting medium containing DAPI. The metaphase images were captured using a confocal microscope (Zeiss LSM 510 Meta; Carl Zeiss) and analyzed with ZEN image software (Carl Zeiss). At least 50 chromosomes were analyzed, and representative images are shown.

### 4.14. Homologous Recombination Assay

HR repair efficiency was measured using the DR-GFP reporter system in U2OS cells as previously described [30]. Briefly, DR-GFP U2OS cells were transfected with the indicated siRNAs using Lipofectamine RNAiMAX (Invitrogen) according to the manufacturer’s instructions. At 12 h post-transfection, cells were transfected with the pCBA-I-SceI plasmid using Turbofect transfection reagent (Thermo Fisher Scientific). After 2 days, the percentage of GFP-positive cells, indicating successful HR repair, was quantified using a BD FACSCalibur flow cytometer (BD Biosciences, San Jose, CA, USA). For each sample, 10,000 events were acquired and analyzed using CellQuest Pro software (BD Biosciences). Data represent mean ± SEM from three independent experiments.

### 4.15. Non-Homologous End Joining Assay

NHEJ assay was measured using the EJ5-GFP reporter system in HeLa cells previously described [30]. Briefly, EJ5-GFP HeLa cells were transfected with control or SLX1 siRNA using Lipofectamine RNAiMAX (Invitrogen) according to the manufacturer’s instructions. At 12 h post-transfection, cells were transfected with the pCBA-I-SceI plasmid TurboFect transfection reagent (Thermo Fisher Scientific). After 36 h, cells were harvested, washed with PBS, and resuspended in flow cytometry buffer. The percentage of hGFP-positive cells, reflecting successful NHEJ repair, was quantified by flow cytometry using a BD FACSCalibur flow cytometer (BD Biosciences, San Jose, CA, USA). For each sample, 10,000 cells were acquired and analyzed using CellQuest Pro software (BD Biosciences). Data represent mean ± SEM from three independent experiments.

### 4.16. Tumor Formation in SCID Mice

The model animals used for this study were 5-week-old male SCID (CB17/lcr-Prkdc^scid/scid^/Rj) mice. These animals were obtained from Janvier Labs (Lille, France) and kept in our pathogen-free animal facility. For the xenograft study, we wanted to determine the role of SLX1 in regulating PARP inhibitor response. Five SCID mice were used per group (total n = 20). An a priori sensitivity analysis (G*Power 3.1; F tests, fixed-effects one-way ANOVA, α = 0.05, power = 0.80, 4 groups, n = 20) indicated a minimum detectable effect size of f ≈ 0.835 (partial η^2^ ≈ 0.41). Therefore, an ethically justified proof-of-concept design with n = 5 per group was employed, as this was sufficiently powered to detect large effects. Data were analyzed using two-way ANOVA with effect sizes and 95% confidence intervals reported. MCF7 cells were harvested and resuspended in PBS. Subsequently, 5 × 10^6^ MCF7 cells were injected subcutaneously into both the left and right sides of the animals. Stratified block randomization based on baseline tumor volume was performed prior to treatment. Before treatment initiation, the tumor size of each mouse was measured with calipers, and the average volume of both flank tumors was defined as the baseline volume. Mice were ranked by baseline tumor volume, and groups of four animals with comparable sizes were formed into randomization blocks. Within each block, mice were randomly assigned to one of four treatment groups (control + vehicle, control + Olaparib, SLX1-knockdown + vehicle, SLX1-knockdown + Olaparib) using a random sequence generated by computer. This ensured equal baseline tumor distribution while maintaining true random allocation. Once the tumors became visible, their sizes were measured at 3 to 4-day intervals using calipers. The tumor volumes were determined according to the formula: Volume = 0.5 × a × b^2^, where ‘a’ and ‘b’ denote the largest and shortest tumor diameters, respectively. All animals were maintained under identical housing conditions (temperature, humidity, and light/dark cycle). Drug administration order was randomized, and tumor measurements and IHC scoring were performed by independent, blinded investigators. About 3 weeks after the injection of the cells, the animals were euthanized, and their primary tumors were excised and immediately weighed.

### 4.17. Bioinformatics Analysis Using the Cancer Genome Atlas (TCGA) Databases

Data from The Cancer Genome Atlas (TCGA; https://www.cancer.gov/about-nci/organization/ccg/research/structural-genomics/tcga, accessed on 15 October 2023) were downloaded using the UCSC Xena browser Data Hub (https://xenabrowser.net/hub/, accessed on 4 December 2023). RNA sequencing data, measured by Illumina HiSeq and normalized with RSEM (RNA-Seq by Expectation-Maximization), were obtained for the breast cancer (TCGA-BRCA) cohort. mRNA expression values were log_2_-transformed for analysis. Correlation analyses between SLX1 expression and clinical parameters were performed and visualized using GraphPad Prism version 9.0 (GraphPad Software Inc., CA, USA). Statistical significance between groups was determined using Student’s *t*-test or Mann–Whitney test, as appropriate. Survival analyses, including overall survival (OS) and disease-free survival (DFS), were performed using Kaplan–Meier curves with log-rank tests via the Human Protein Atlas database (https://www.proteinatlas.org/, accessed on 9 May 2024). Genomic alteration frequency analysis was performed using the cBioPortal for Cancer Genomics (https://www.cbioportal.org, accessed on 5 August 2024), which provides interactive exploration of cancer genomics data. We systematically analyzed SLX1 alteration frequency and type in the pan-cancer cohort.

### 4.18. Ethics Statement

All animal experiments were performed in accordance with the ARRIVE 2.0 guidelines (Animal Research: Reporting of In Vivo Experiments) and approved by the Institutional Animal Care and Use Committee (IACUC) of Chosun University School of Medicine (CIACUC2023-A0028).

### 4.19. Statistical Analysis

All data are presented as the mean ± SEM from at least three independent experiments unless otherwise indicated. Statistically significant differences between groups were determined using a two-tailed paired Student’s *t*-test. For multiple group comparisons, one-way or two-way ANOVA was performed. All statistical analyses were performed using GraphPad Prism 9.0 (GraphPad Software Inc., San Diego, CA, USA). Results with a value of * *p* < 0.05, ** *p* < 0.01, and *** *p* < 0.001 were considered statistically significant.

## Data Availability

The datasets used in this study are publicly available. The RNA sequencing data and clinical information for the 12 TCGA cancer types (BRCA, BLCA, ESCA, GBM, KICH, KIRC, KIRP, LUAD, LUSC, HNSC, PRAD, and THCA) were obtained via the UCSC Xena browser Data Hub (https://xenabrowser.net/hub/, accessed on 4 December 2023) from The Cancer Genome Atlas (TCGA; https://www.cancer.gov/about-nci/organization/ccg/research/structural-genomics/tcga, accessed on 15 October 2023). Genomic alteration frequencies were analyzed using the cBioPortal platform (www.cbioportal.org, accessed on 5 August 2024) based on a pan-cancer cohort.

## Supplementary Materials

The following supporting information can be downloaded at https://www.mdpi.com/article/10.3390/ijms262311621/s1.
