# Supplementary material for: SLX1 Inhibition Enhances Olaparib Sensitivity by Impairing Homologous Recombination Repair in Breast Cancer
Source: Int J Mol Sci. 2025 Nov 30;26(23):11621. doi: 10.3390/ijms262311621 (PMC12692287; doi:10.3390/ijms262311621)
Supplement: Supplementary file 1 [file ijms-26-11621-s001.zip › ijms-3958256-supplementary.pdf]

Supplementary Materials

7 supplementary figures

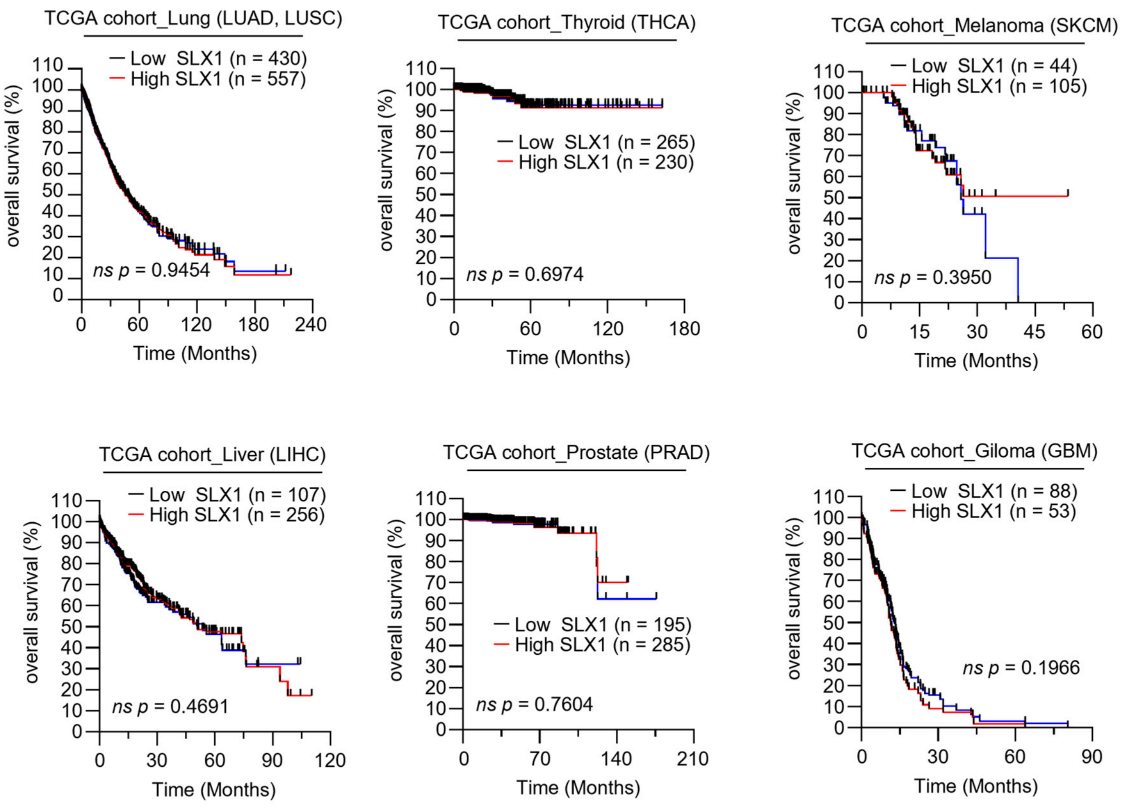

Supplementary Figure S1. SLX1 levels are not indicative of overall survival for cancers other than breast cancer. Kaplan–Meier analysis of overall survival based on SLX1B expression in Lung, Thyroid, Melanoma, Liver, Prostate and Glioma cancers using TCGA cohort data.

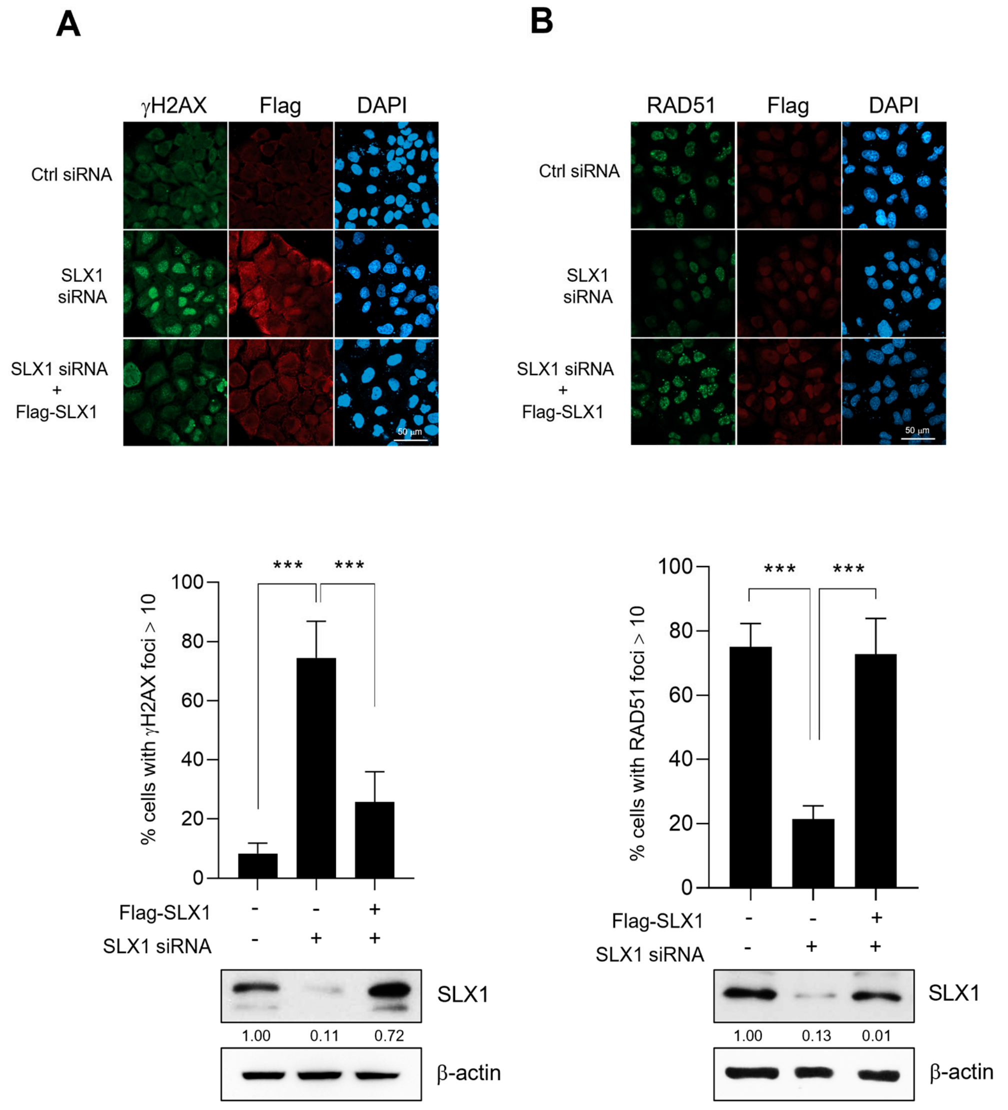

Supplementary Figure S2. Restoration of SLX1 rescues γH2AX and RAD51 foci formation following ionizing radiation. (A) HeLa cells are transfected with SLX1 siRNA and rescued with Flag-SLX1 expression. Cells are exposed to 10 Gy ionizing radiation and immunostained after 20 hours to detect γH2AX foci. Histogram shows the percentage of cell populations cells with more than ten γH2AX foci. SLX1 expression levels are confirmed by western blot. Scale bar = 50 µm. Data are presented as mean ± SEM. ***$p < 0.001$, two-tailed Student's t-test. **(B)** Similarly treated HeLa cells are analyzed 6 hours post-irradiation to detect RAD51 foci. The number of RAD51 foci per cell is shown in the histogram (n = 3). SLX1 expression is validated by western blot. Scale bar = 50 µm. Data are mean ± SEM. ***$p < 0.001$, two-tailed Student's t-test. All experiments are independently repeated three times.

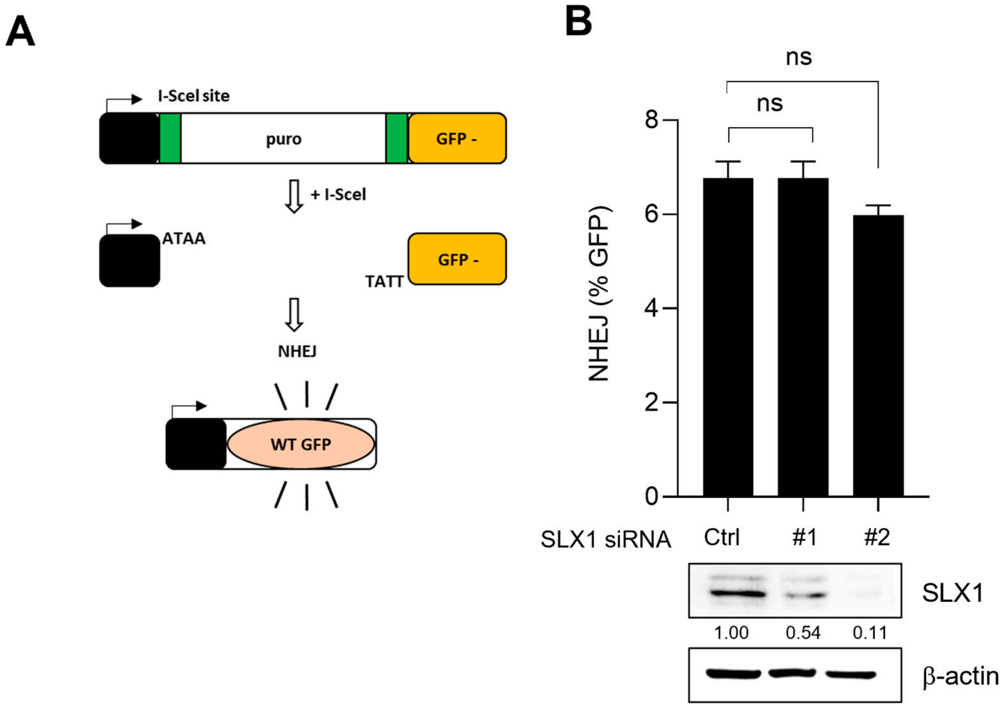

Supplementary Figure S3. SLX1 depletion does not impair non-homologous end joining (NHEJ) repair efficiency. (A) Schematic of the fluorescence-based assay using an EJ5-GFP reporter system to measure NHEJ-mediated DSB repair. **(B)** HeLa cells expressing the EJ5-GFP reporter are transfected with control or SLX1B siRNA, and NHEJ efficiency is measured using FACS. SLX1B expression is confirmed by western blot. Data are shown as mean ± SEM (n = 3). ns = not significant. All experiments are independently repeated three times.

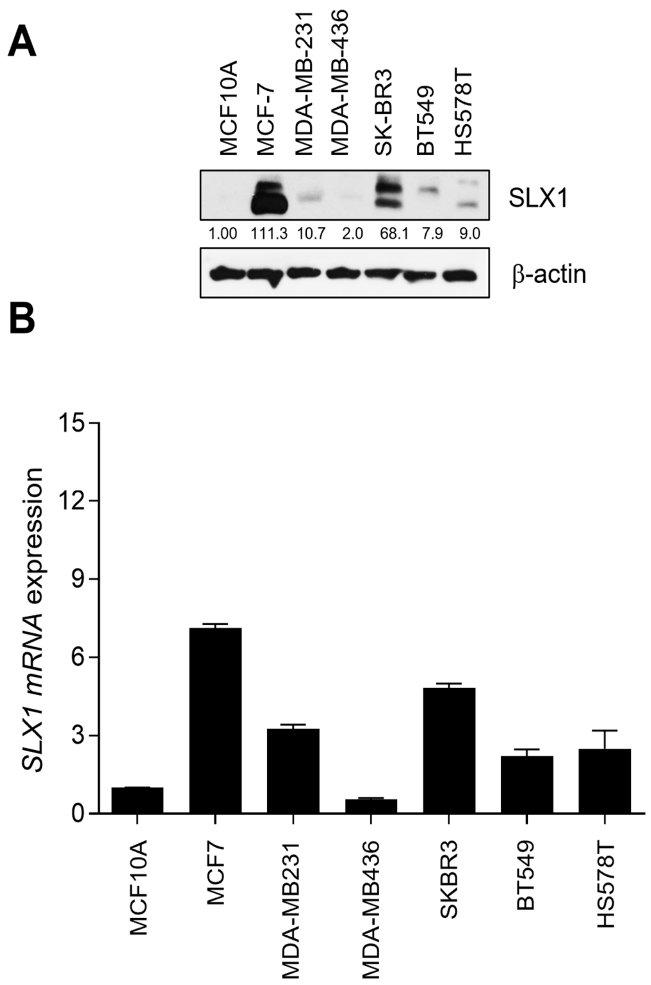

Supplementary Figure S4. SLX1 is aberrantly upregulated in breast cancer cell lines. Protein lysates from normal breast epithelial cells (MCF10A) and breast cancer cell lines (MCF7, MDA-MB231, MDA-MB436, SKBR3, BT549, and HS578T) are harvested and lysed. **(A)** Immunoblotting analysis is performed using specific antibodies against the corresponding proteins. The Figure is representative of at least three separate experiments that yielded similar results. **(B)** SLX1 mRNA expression is analyzed using qRT-PCR and graph represented mean ± SEM (n = 3). All experiments are independently repeated three times.

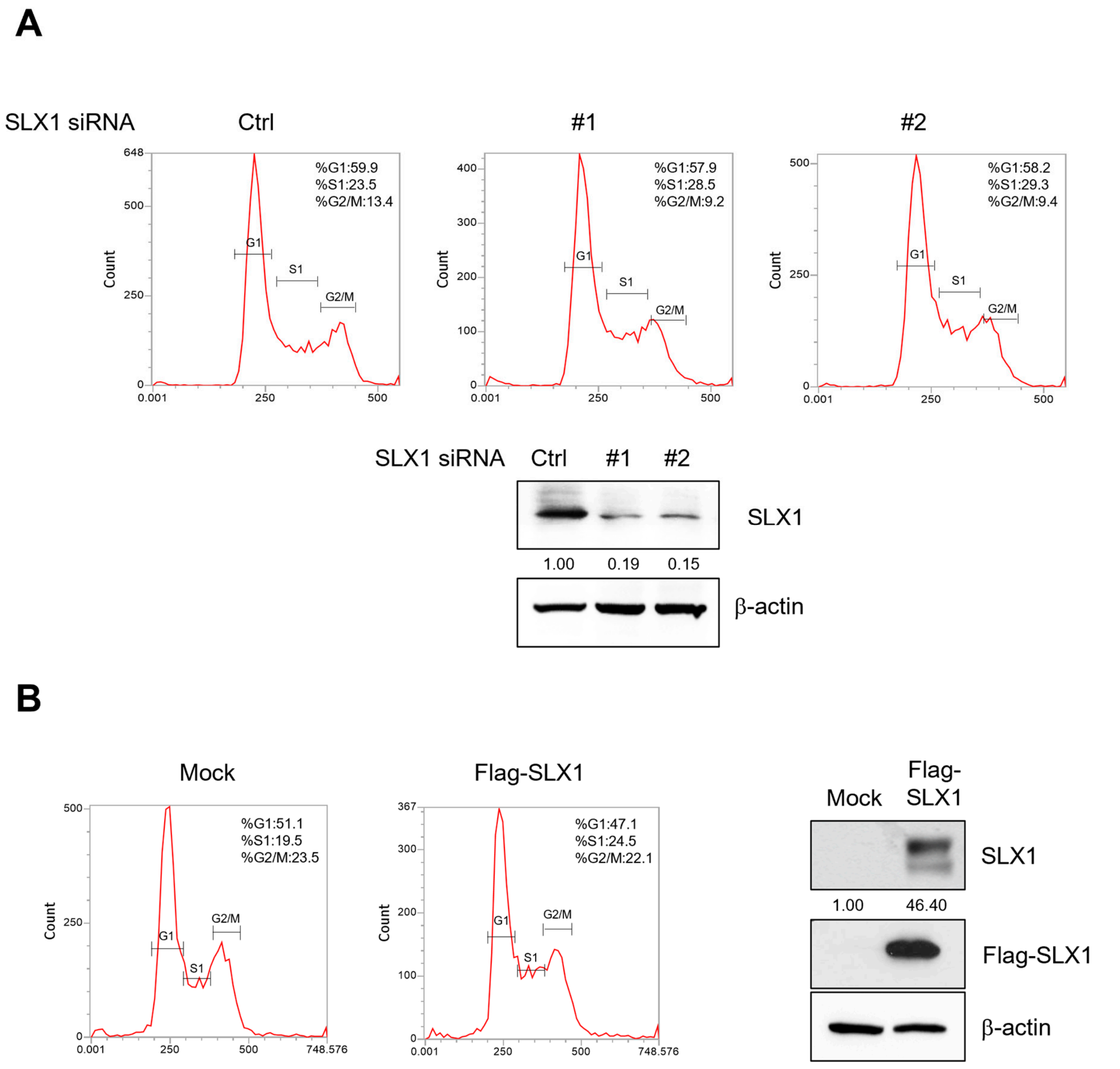

Supplementary Figure S5. The level of SLX1 expression is independent of the cell cycle distribution. (A, B) Control and SLX1-knockdown MCF7 cells (**A**), as well as MDA-MB231 cells transfected with either a control vector (Flag-mock) or SLX1-overexpressing construct (Flag-SLX1) (**B**), are measured the cell cycle distribution by Flow cytometry analysis. SLX1 knockdown or overexpression is confirmed by western blot. All experiments are independently repeated three times.

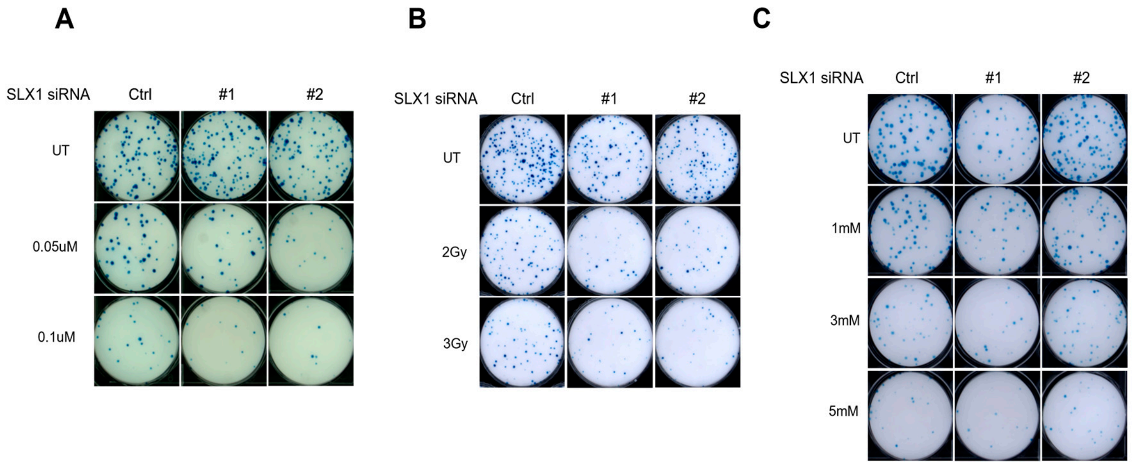

Supplementary Figure S6. SLX1 affects cell viability following DSBs. (A, B, C) Representative colony images correspond to Figures 3E**(A)**, 3F**(B)**, and 3G**(C)**, respectively. These images show the morphology and cell count after colony formation is halted. All experiments are independently repeated three times.

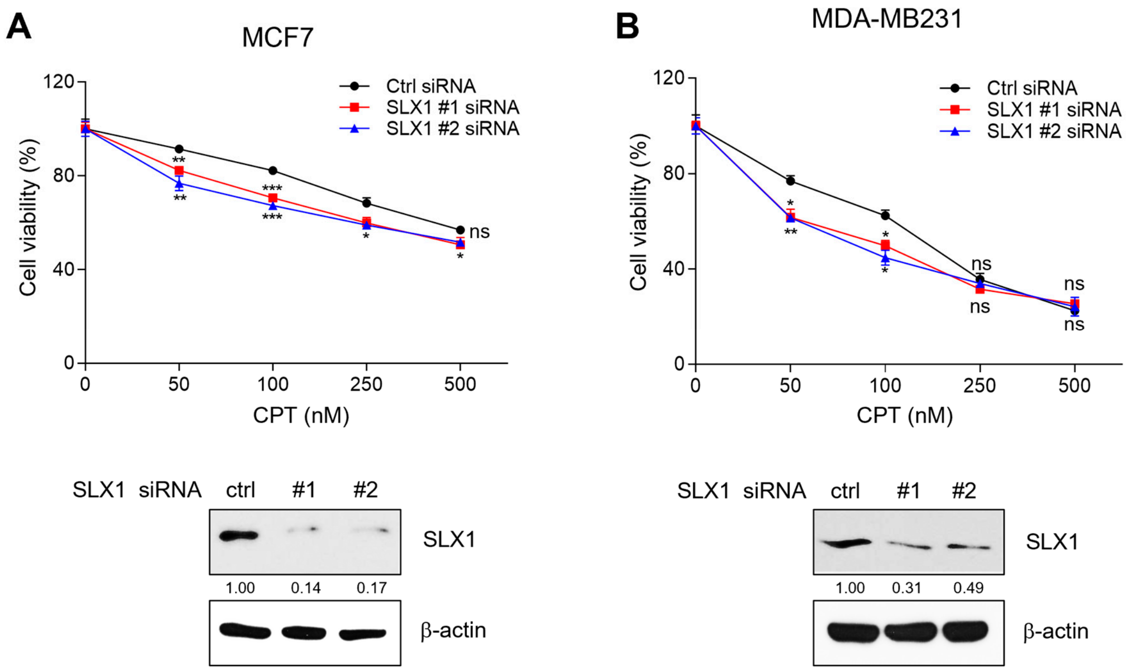

Supplementary Figure S7. SLX1 weakly reduces cell viability to CPT in breast cancer cells. (A, B) MCF7 (**A**) and MDA-MB231 (**B**) cells are transfected with either control siRNA or SLX1-specific siRNAs. After 24 hours, cells are treated with CPT for 48 hours, and cell viability is then assessed using the MTT assay. Graph are normalized to the non-treated sample and shown as relative cell viability (%). Data are presented as mean ± SEM. *$p < 0.05$, **$P < 0.01$, ***$p < 0.001$. ns, not significant. Western blots showed SLX1 levels in cells used in these experiments. All experiments are independently repeated three times.
